# 8-Oxoguanine: A Lesion, an Epigenetic Mark, or a Molecular Signal?

**DOI:** 10.3390/ijms262411799

**Published:** 2025-12-06

**Authors:** Anton V. Endutkin, Antonina P. Dvornikova, Dmitry O. Zharkov

**Affiliations:** 1 Institute of Chemical Biology and Fundamental Medicine, Siberian Branch of the Russian Academy of Sciences, 8 Lavrentieva Ave., 630090 Novosibirsk, Russia; antdovgerd@1bio.ru; 2 Department of Natural Sciences, Novosibirsk State University, 2 Pirogova St., 630090 Novosibirsk, Russia

**Keywords:** DNA damage, DNA repair, gene expression, 8-oxoguanine, OGG1

## Abstract

For decades, 8-oxoguanine (8-oxoG) has been recognized as a pervasive and pro-mutagenic oxidative DNA lesion. In human cells, 8-oxoG is removed from DNA via the base excision repair pathway initiated by 8-oxoguanine–DNA glycosylase (OGG1). However, emerging evidence over the past twenty years suggests a more complex, regulatory role for this DNA modification. Here, we discuss findings that 8-oxoG, particularly when present in gene promoters, can act as a signal to modulate transcription, establishing an 8-oxoG/OGG1 axis in the inflammatory response. Proposed mechanisms include the generation of 8-oxoG during chromatin remodeling processes involving histone demethylases, the recruitment of transcription factors (NF-κB, HIF1α, Myc, SMAD, etc.) by OGG1, and the lesion’s enrichment in guanine-rich sequences prone to forming G-quadruplex structures. The pro-mutagenic nature of 8-oxoG and the lack of dedicated, functionally separate writer and reader proteins challenge its classification as a true epigenetic DNA mark, distinguishing it from canonical epigenetic nucleobases like 5-methylcytosine and 5-hydroxymethylcytosine. On the other hand, 8-oxoG is well suited for the role of a regulatory signal localized to DNA and involved in the cellular response to oxidative stress and the associated physiological stimuli.

## 1. Introduction: 8-Oxoguanine and OGG1

**Figure 1:**
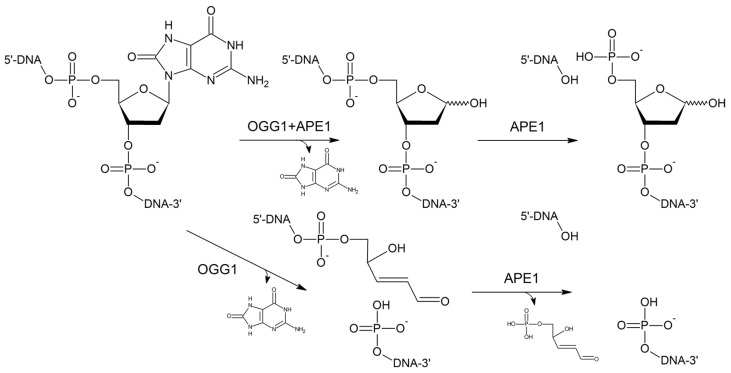
Structure of 8-oxoG and the mechanisms of its repair involving OGG1 DNA glycosylase and APE1 AP endonuclease.

Reactive oxygen species generated during oxidative stress readily oxidize proteins, lipids, and nucleic acids. Oxidation of genomic DNA bases can lead to mutations responsible for a number of diseases [1,2,3]. Of all nucleobases, guanine is the most sensitive to oxidation, which produces more than a dozen products [4]. Of these, 8-oxoguanine, discovered in early 1980s [5], is formed in the largest amount and is, therefore, often used as a biomarker to assess the level of oxidative stress to which cells or the organism as a whole have been exposed [6,7]. If persisting in DNA until replication, 8-oxoG can form Hoogsteen pairs with A and serve as a precursor of mutations, mainly G→T transversions, which significantly contribute to the initiation and progression of many cancers [8,9,10,11]. To counteract these deleterious consequences, a three-tier DNA repair mechanism known as the GO (guanine oxidation) system evolved within the BER pathway [12]. In human cells, the removal of 8-oxoG from 8-oxoG:C pairs is initiated by 8-oxoguanine–DNA glycosylase (OGG1; Figure 1). On the contrary, when 8-oxoG is opposite A, MUTYH DNA glycosylase, the second member of the GO system, removes the adenine base, allowing DNA polymerase to incorporate C opposite 8-oxoG for further processing by OGG1. The third player in the GO system, MTH1, is a nucleoside triphosphate hydrolase that sanitizes cellular dGTP pool converting 8-oxodGTP to 8-oxodGMP. After the removal of 8-oxoG by OGG1, an apurinic/apyrimidinic site (AP site) is formed, which is cleaved by AP endonuclease (APE1; Figure 1). The repair process is completed by DNA polymerase β and DNA ligase IIIα. It is estimated that oxidation of G to 8-oxoG and subsequent repair occurs in each human cell up to 10^5^ times per day [13,14]. Along with 8-oxoG, oxidation of G generates other lesions, such as 2,4-diamino-6-oxo-5-formamidopyrimidine, guanidinohydantoin and spiroiminodihydantoin, which are produced in slightly lower but still noticeable amounts [4,15]. These lesions are removed predominantly by NEIL1 DNA glycosylase, which also shows some activity towards 8-oxoG.

**Figure 2:**
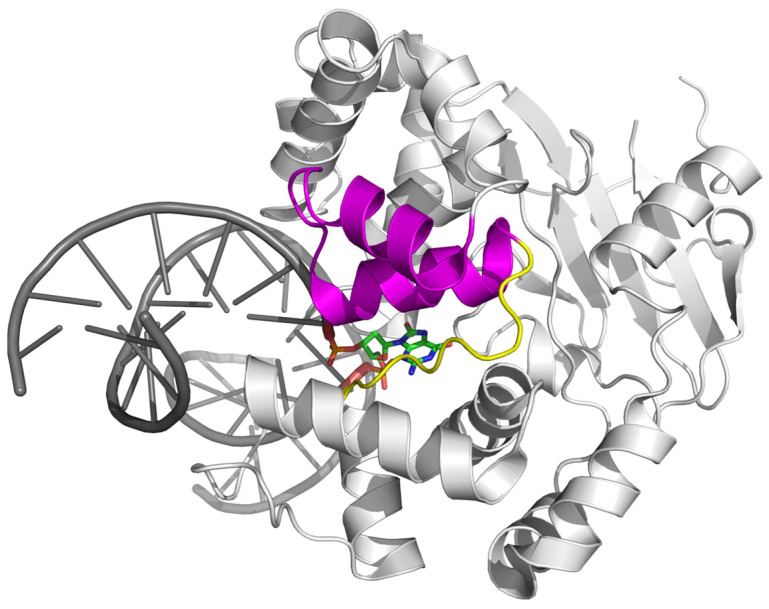
Structure of human OGG1 bound to damaged DNA (PDB ID 1EBM [35]). The HhH motif is colored magenta, the Gly/Pro/Val-rich loop, yellow. 8-oxoG is shown as a stick model.

OGG1 is one of eleven known human DNA glycosylases [16,17]. OGG1 belongs to the large helix–hairpin–helix (HhH) structural superfamily, which includes the proteins containing the HhH non-specific DNA binding motif [18,19] (Figure 2). DNA glycosylases from the HhH superfamily additionally contain a Gly/Pro/Val-rich loop terminating with the absolutely conserved catalytic Asp residue (Figure 2). Their substrate specificity varies widely; in addition to OGG1, human cells contain three more HhH DNA glycosylases: NTHL1, specific for oxidized pyrimidines, MBD4, removing deaminated cytosine and 5-methylcytosine from CpG dinucleotides, and MUTYH, which, as mentioned above, excises A opposite to 8-oxoG [16,20]. OGG1 is the main 8-oxoG DNA glycosylase in humans and additionally can remove 2,4-diamino-6-oxo-5-formamidopyrinidine, another product of oxidative damage to G [21,22]. A characteristic feature of OGG1 is its slow turnover: after 8-oxoG excision, the enzyme stays bound to its AP site product with the half-life of 10–20 min but can be displaced by APE1, the next enzyme in the BER pathway [23,24,25,26,27]. This sequence of events produces a single-stranded break with a 5′-terminal 2′-deoxyribose-5′-phosphate remnant (Figure 1, top pathway). However, if OGG1 has enough time, it cleaves AP site by β-elimination, leaving a 3′-terminal α,β-unsaturated aldehyde moiety, which requires removal by APE1 to yield a one-nucleotide gap flanked by 3′-OH and 5′-phosphate groups (Figure 1, bottom pathway). The tight OGG1–product complex is not only necessary for keeping highly reactive AP sites from forming stand breaks and poorly repaired adducts [28] but also provides ample possibilities for organizing regulatory complexes around the sites of oxidative damage [29,30,31,32,33,34]. However, the architecture of these macromolecular associates is not known at present.

Until recently, the prevailing opinion based on the demonstrated pro-mutagenic role of 8-oxoG in replication was that this modified nucleobase is solely a malicious lesion in need of prompt repair. However, numerous studies over the past twenty years argued that 8-oxoG may be an important cellular signal regulating the activity of at least some genes in response to the redox balance in the cell, especially in connection with inflammation. It has been even claimed sometimes that 8-oxoG is a new epigenetic base [36,37,38]. Here, we discuss the evidence for the role of 8-oxoG as a molecular signal and its place among other oxidative stress-related regulatory pathways.

## 2. Epigenetic DNA Bases

Epigenetic mechanisms are responsible for the transmission of information about the state of a cell through its division (both for unicellular organisms and individual cells of multicellular organisms), or, in some cases, even across generations in multicellular sexually reproducing organisms. Gene activity in eukaryotes is largely regulated by the interaction between RNA polymerases bound at the promoter regions and a variety of transcription factors and genomic regulatory elements (enhancers and silencers) [39,40]. Active promoters are mainly characterized by an open state of chromatin, which allows transcription factors to bind their recognition sites in DNA. Of many mechanisms regulating stable patterns of eukaryotic gene expression, two are best studied to date. The first one is associated with the state of chromatin condensation due to covalent modification of histones or other proteins responsible for DNA packaging. The second one is based on covalent modification of DNA itself, which regulates the affinity of various DNA-binding proteins for their target sequences. Both mechanisms play key roles in development of multicellular eukaryotes, transcriptional response to external stimuli, adaptation to the environment, and many pathological processes, and their states can be carried through cell division [41,42,43,44].

**Figure 3:**
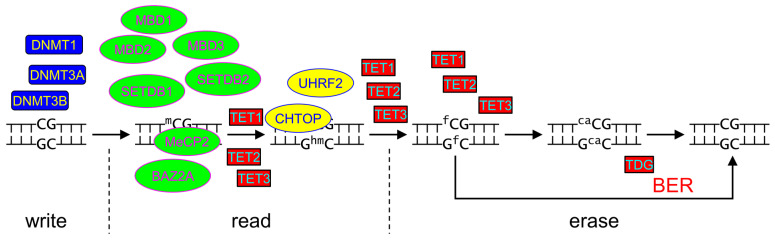
Functional separation between DNA modification writers (blue), readers (green and yellow) and erasers (red) in the mC-mediated epigenetic regulation system. Differences between fully methylated and hemimethylated CpG dinucleotides are omitted for clarity.

At present, the most thoroughly investigated epigenetic DNA modifications in vertebrate genomes are 5-methylcytosine (mC) and, more recently, 5-hydroxymethylcytosine (hmC) (Figure 3, Table 1). For DNA methylation, mammalian cells possess three DNA methyltransferases, DNMT1, DNMT3A, and DNMT3B, which transfer a methyl group from *S*-adenosylmethionine to C5 of the cytosine base in CpG dinucleotides [45]. DNMT3 enzymes are required for the establishment of DNA methylation de novo, while DNMT1 maintains the methylation status by recognizing hemimethylated CpG dinucleotides and modifying C in the daughter strand after replication. Active demethylation is carried out by TET family dioxygenases (TET1, TET2, and TET3 in humans), which oxidize mC to hmC and further to 5-formylcytosine (fC) and 5-carboxylcytosine (caC) [46,47,48]. Both fC and caC are then removed from DNA by mismatched thymine–DNA glycosylase (TDG) with the BER pathway ultimately reinstalling unmodified C [47,49,50,51].

Cytosine methylation in promoters often leads to gene repression, but mC within transcribed sequences, on the contrary, often marks active genes [52]. Thus, mC is one of the main regulators of transcription at the level of DNA chemistry. The effect of DNA methylation on gene activity is mainly mediated by mC binding to “reader” proteins that specifically recognize it and either possess enzymatic activity themselves (e.g., histone methyltransferases SETDB1 and SETDB2 or deacetylase BAZ2A) or can recruit other enzymes, transcription factors, and chromatin remodeling factors (methyl-CpG-binding proteins MeCP2, MBD1, MBD2, MBD3, etc.; Figure 3). In contrast to mC, hmC is enriched in the promoter regions of actively transcribed genes and is considered an activating epigenetic mark [53,54]. Proteins that specifically bind hmC in DNA have been discovered [55,56,57], but there is still no mechanistical understanding of how exactly this modification affects transcriptional activity.

The example of C5-modified cytosine clearly illustrates important features of epigenetic marks in DNA. They are (i) introduced and (ii) erased in a regulated manner by specific enzymes and (iii) are interpreted by dedicated readers (Figure 3). Moreover, the persistence of epigenetic labels in DNA over many generations require them (iv) to be easily bypassed by DNA polymerases in an error-free manner. It is currently unclear whether other eukaryotic DNA modifications possess these features. For example, deamination of hmC by cytidine deaminases of the AID/APOBEC family generates 5-hydroxymethyluracil (hmU) in DNA [50,58]. In addition, thymine, like mC, contains a methyl group at position C5 and can be oxidized with some efficiency by TET enzymes to hmU in vivo [59]. Moreover, in some protozoans, hmU is specifically produced through enzymatic oxidation of T by JBP family dioxygenases, homologs of vertebrate TET proteins, and serves as an intermediate in the synthesis of β-D-glucosylhydroxymethyluracil (“Base J”), which plays a role in transcriptional regulation in these organisms [60,61,62]. Finally, hmU is recognized by MeCP2, a known epigenetic mark reader [59], and promotes DNA demethylation at a distance of up to several hundred nucleotides from the site of origin through combined action of base excision repair and mismatch repair [63]. However, due to the impossibility to distinguish between hmU generated by targeted and spontaneous deamination, limited experimental data on the mechanisms of hmU formation, its distribution in the genome, its effect on gene activity, proteins that recognize it, and its prevalence in nature, this modified base is generally not considered epigenetic or regulatory. Another non-cytosine base with a suspected epigenetic role is 6-methyladenine (mA). It is widespread in the genomes of bacteria, archaea, and protozoa [64], but was also reported to be found at low levels in DNA of plants, nematodes, insects, and vertebrates, including humans [65,66,67,68,69]. Transcription termination sites, promoters and intergenic regions are enriched in mA. Specific adenine *N*^6^-methyltransferases N6AMT1, METTL3-METTL14 and METTL4, oxidative mA demethylases ALKBH1 and ALKBH4, and potential specific readers ASXL1, MPND and YTHDC1 have been identified in mammals [68,70,71,72,73]. Also, mA is apparently tolerated during DNA replication, although it moderately inhibits human translesion DNA polymerases η and ι [74,75]. Yet, the low abundance of mA in DNA of higher eukaryotes and the ongoing debate of the technical pitfalls and artifacts in its detection [76,77] so far preclude definite classification of mA as an epigenetic base.

It should be emphasized that the concepts of “epigenetic” and “regulation of gene activity” are not equivalent: the former implies the establishment of expression patterns that are stable for a sufficiently long time and maintained even after cell division, while the latter can be quite short-term and dynamic. It should also be taken into account that targeted DNA modifications in higher organisms can play not only a regulatory role but also initiate other processes. For example, it is well known that somatic hypermutation and class switch recombination in immunoglobulin genes is induced by regulated deamination of C to uracil in DNA, followed by the recruitment of repair enzymes and specialized DNA polymerases [78,79]. The question of the potential epigenetic function of other modified DNA bases—or even simply their biological role in regulating gene activity—currently remains open.

## 3. Is 8-Oxoguanine Regulatory?

### 3.1. OGG1 Binds 8-oxoG in Promoters and Recruits Transcription Factors

The idea of a possible epigenetic/regulatory role for 8-oxoG appeared after the presence of this lesion in the promoters of some genes, such as *BCL2*, *SIRT1*, *TNF* and *VEGF*, was found to be associated with their activation [80,81,82,83,84]. Since then, several hypotheses have been put forward about the possible mechanisms of this activation.

In some cases, an increase in the affinity of the damaged promoter for known transcription factors was found: for example, 8-oxoG in specific positions of the sequence recognized by the p50 subunit of the NF-κB transcription factor leads to better binding [85], and oxidation of the *VEGF* promoter stimulates transcription due to increased binding of Sp1 [80,86]. However, in most cases, DNA damage is detrimental for target recognition by sequence-specific proteins [85,87,88,89], and 8-oxoG-dependent recruitment of transcription factors is indirect. For example, hypoxia-inducible genes, including *VEGF*, recruit OGG1 and APE1 to their oxidized promoters, which facilitates binding of HIF1α to the hypoxia responsive element and enhances transcription [83]. Appearance of 8-oxoG in the NF-κB recognition site in the promoters of TNFα-responsive genes causes OGG1 binding, which in turn induces NF-κB RelA/p65 binding and phosphorylation, resulting in transcription activation [84,90,91,92,93]. In an analogous manner, OGG1 binds oxidized DNA near the sequences recognized by Myc and several members of the SMAD family and recruits these transcription factors to their promoters [94,95,96,97]. Interactions of OGG1 with other transcription factors or co-factors, such as MAZ, HNRNPA1, and PPARγ have also been reported but its functional consequences have not been established yet [98,99]. Finally, OGG1 can recruit PRMT1 arginine methyltransferase, which produces an activating histone mark, asymmetrically methylated arginine H4R3me2a [100].

One unresolved question is how the signal can be maintained after OGG1 has excised 8-oxoG from DNA. The excision would create problems for the stability of the complex given the high abundance of APE1 [101], which releases OGG1 from its product [23,24,25,26,27]. It is possible that the enzyme binds 8-oxoG in non-canonical DNA structures not permissive for catalysis, such as G-quadruplexes (see Section 3.3 below). Another possibility is that the regulatory role is played by enzymatically inactive OGG1, which is proficient in stimulating transcription [102]. Wild-type OGG1 and, to an even greater extent, its cancer-associated variant OGG1 S326C are inactivated through oxidation of critical Cys residues by physiological inflammation-induced levels of ROS and by the free radical cellular messenger, NO [103,104,105,106]. For OGG1 S326C, it was also suggested that oxidation of Cys326 promotes covalent dimerization, which enhances OGG1 binding to DNA even if the protein remains catalytically active [107]. 4-Hydroxy-2-nonenal, a product of lipid damage by ROS, also inactivates OGG1 reacting with Cys, Lys, and His side chains [108]. Ultimately, however, transcription activation may be mediated not by OGG1 itself but by its interaction partner, APE1, through the redox domain (Ref-1) of the latter. This domain reduces and re-activates a number of spuriously oxidized transcription factors, including AP-1, NF-κB, Myb, HIF1α, and p53 [109,110,111,112,113,114]. Moreover, post-translationally modified APE1 binds some transcription factors, such as YB-1, even when they are not oxidized, thereby assisting RNA polymerase II loading onto promoters [115,116,117]. On the other hand, oxidative damage-related activation of *SIRT1* expression involves the assembly of complexes including OGG1, APE1 and the DNA break sensor Ku70, suggesting that at least in this case the repair function of APE1 is required [82]. Inhibition of both DNA repair and redox activity of APE1 downregulates the expression of pro-inflammatory cytokines (see Section 3.5 below) [118].

In some situations, 8-oxoG can also negatively regulate transcription. A mode of regulation similar to that described above but of inhibitory nature was observed for interferon λ2/λ3 promoters where OGG1 binds and recruits NF-κB p50/p50 transcription repressor complexes [119]. In some cases, however, OGG1 simply competes with transcription factor binding, as was shown for the CREB1 target site [120]. Intermediates of 8-oxoG repair in the promoter regions and in the non-template DNA strand also strongly impair transcription [121,122,123], while the presence of 8-oxoG in the DNA template causes RNA polymerase II to pause [124]. Moreover, OGG1 binds to 8-oxoG in the DNA:RNA heteroduplex in R-loops and inhibits transcription [125]. Oxidative damage to DNA also triggers histone deacetylation in nucleosomes around the promoter concomitant with transcription suppression [126].

### 3.2. 8-oxoG Can Be Generated in the Process of Chromatin Remodeling

One widely discussed hypothesis for the relationship between 8-oxoG formation and transcription activation is centered on histone demethylation followed by chromatin remodeling. According to this model, DNA oxidation is induced by reactive oxygen species generated in situ during oxidative demethylation, and then OGG1 binding initiates transcription activation. This mechanism was proposed for the genes regulated by estrogen receptor (ER) in breast cancer cells [81]. Estrogen-bound ER translocates to the nucleus where it recognizes its target sequences and activates lysine demethylase 1A (LSD1) to remove the repressive H3K9me2 marks. LSD1 oxidizes *N*,*N*-dimethyllysine using FAD as an electron acceptor that is then re-oxidized by O_2_ with H_2_O_2_ formed as a byproduct [127]. Thus, 8-oxoG can be generated in a close vicinity of demethylated histones and attract OGG1, which in turn recruits transcription factors. As an additional way of transcription activation, if the repair proceeds beyond 8-oxoG removal and APE1 cleaves DNA next to the AP site, topoisomerase IIβ can be loaded onto the break, ultimately leading to chromatin remodeling.

In addition to the ER-regulated genes, the LSD1→H_2_O_2_→8-oxoG→OGG1 pathway was proposed to operate in the activation of gene expression by the androgen receptor [128], retinoic acid receptor [129] and Myc [130]. The histone mark removed may be either H3K4me2 or H3K9me2, and LSD1 was implicated in all cases. A more complex two-step mechanism was suggested for TGF-β1-induced gene regulation during epithelial–mesenchymal transition [131]. Initially, TGF-β1 triggers phosphorylation of SMAD2/3 transcription factor, which activates LSD1 and another oxidative histone demethylase, JMJD2A. OGG1-mediated transcription of the target genes ensues, producing, among others, SNAI1 repressor protein. Later, SMAD-activated LSD1/JMJD2A continue to generate 8-oxoG and attract OGG1 but this time to assemble SNAI1-containing complexes on a set of promoters repressed during epithelial–mesenchymal transition.

Fe^II^/α-ketoglutarate-dependent TET dioxygenases, which oxidize mC to hmC, fC and caC, are another possible source of leaking ROS that could produce 8-oxoG [132]. So far, however, there is only circumstantial evidence that TETs could regulate transcription in this way, and it would be extremely hard to distinguish any effect from the regulation through active DNA demethylation. On the contrary, OGG1 can recruit TET1 and TET2 to the sites of 8-oxoG damage and promote demethylation (see Section 3.4 below) [97,133].

In all these cases, it remains unclear to what extent the ROS flux from the oxidative chromatin demethylation reactions is sufficient to form noticeable amounts of 8-oxoG and how specific is the formation of 8-oxoG compared to other types of DNA damage. Pharmacological inhibition of LSD1 reduces the expression of the *Tnf* target gene in wild-type mice but not in *Ogg1* knockouts suggesting that LSD1 and OGG1 are epistatic in vivo [134] but still leaving many options for the possible mechanism.

### 3.3. Potential Quadruplex-Forming Sequences Often Found in Gene Promoters Are Hotspots for G Oxidation

**Figure 4:**
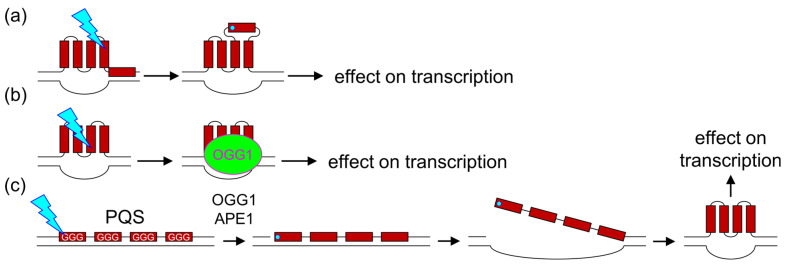
Possible mechanisms of G-quadruplex-mediated 8-oxoG-dependent transcription regulation. (**a**) Oxidation of G in a pre-existing quadruplex followed by quadruplex refolding with a previously unengaged G-run (“spare tire” model). (**b**) Oxidation of G in a pre-existing quadruplex followed by OGG1 binding in a catalytically incompetent mode. (**c**) Oxidation of G in a PQS followed by OGG1-, APE1-dependend nicking leading to duplex destabilization and G-quadruplex folding. 8-oxoG is shown as a cyan dot, G-runs as red rectangles.

A currently popular hypothesis about the mechanism of 8-oxoG effect on gene transcription is that it could be mediated by DNA topology, in particular, via G-quadruplex formation. It is known that G-runs, especially their first and second positions, are the most easily oxidized sites in duplex DNA [135,136]. Even if the primary loss of electron occurs at some other place, the hole migrates along the base stack until it settles on the G-run [137,138]. Thus, potential quadruplex-forming sequences (PQSs), which possess several juxtaposed G-runs, are natural hotspots for G oxidation. PQSs are often found in gene promoters, including those of the genes induced by oxidative stress, such as *VEGF*, *NTHL1*, *HRAS*, *KRAS*, *MT1A*, *NFKB2* or *PCNA*, and accumulate elevated levels of 8-oxoG [98,139,140,141,142]. Once formed, 8-oxoG alters the topology of the G-quadruplex since it cannot form the N7…HN^2^ hydrogen bond characteristic of the tetrad. This can lead to quadruplex unfolding [143,144,145,146] or refolding to a variety of different structures if a spare undamaged G or several Gs are available (“spare tire” model) [98,147] (Figure 4a).

Several possible mechanisms of 8-oxoG-dependent, quadruplex-mediated gene activation have been proposed. In the simplest variant, OGG1 can bind 8-oxoG in a pre-existing G-quadruplex in a catalytically incompetent mode so the enzyme does not excise the lesion but still attracts transcription factors [98] (Figure 4b). If 8-oxoG arises in a PQS in a DNA duplex, OGG1 can remove 8-oxoG producing an AP site, which destabilizes the duplex and promotes its refolding to a quadruplex (Figure 4c). APE1 then binds the AP site in the quadruplex but does not cleave it, remaining in the promoter and activating transcription factors through the redox function of its Ref-1 domain [141,148]. Alternatively, APE1 might cleave the AP site but remain tethered to the quadruplex through its positively charged unstructured N-terminal tail, which can be acetylated at several Lys residues to release DNA in a regulated manner [148,149]. It has been shown that G-quadruplex refolding can occur in cellulo with the participation of OGG1 and APE1 [148]. In contrast, formation of 8-oxoG in the G-quadruplex without damaged base excision represses transcription, as has been shown for the *VEGF*, *NEIL3*, and *RAD17* promoters [150,151,152], possibly due to the quadruplex topology changes, as discussed above.

In addition to G-quadruplexes, 8-oxoG removal from cruciform structures, followed by their refolding, can affect transcription either positively or negatively depending on the position of 8-oxoG [153]. However, gene promoters are not enriched in inverted repeats potentially forming cruciform structures, and these sequences do not feature prominent G-runs, so the biological relevance of this mechanism remains obscure.

### 3.4. 8-oxoG Can Affect DNA Methylation Status Either Directly or Through OGG1

The possible influence of 8-oxoG on mC- and hmC-dependent epigenetic processes has not been ignored. In regard to active methylation, oxidation of G in CpG sites reduces their affinity for DNMT enzymes, suppressing both de novo and maintenance methylation [154,155]. When G is oxidized in methylated CpG sites, the resulting mCp(8-oxoG) dinucleotides are not recognized by the repressive mC reader, MeCP2 [156]. No data for other readers in human cells is available but partial inhibition of DNA cleavage opposite 8-oxoG was shown for plant ROS1 epigenetic 5-methylcytosine–DNA glycosylase [157]. Given that 8-oxoG destabilizes DNA duplex [158] and alters the dynamics of mCpG sites [157,159], and that MBD family proteins recognize the N7 position of G that changes from a hydrogen bond acceptor to a donor in 8-oxoG [160,161,162,163], the effect on other mC-recognizing proteins is quite possible.

Regulation of DNA methylation mediated by OGG1 has also been reported. For example, OGG1 recruits chromodomain helicase DNA binding protein 4 (CHD4) and enhancer of zeste homolog 2 (EZH2) to sites of 8-oxoG formation in promoters of tumor suppressor genes [34]. CHD4 is a chromatin remodeler that has histone deacetylase activity and also loads all DNMTs onto DNA inducing both de novo and maintenance methylation, while EZH2 is a histone methylase [34]. In a recent work, both OGG1 and its partner in the GO system, MUTYH, were found to modulate the assembly of Polycomb repressive complexes on gene promoters, although the association with 8-oxoG was not addressed [164]. MUTYH-deficient familial colorectal adenomas are globally hypomethylated compared with MUTYH-proficient ones, supporting a possible role of MUTYH in addition to OGG1 in the regulation of mC epigenetic status [165]. The appearance of 8-oxoG in DNA contributes to the suppression of expression of damaged regions by inducing CpG islands methylation and chromatin silencing [166,167]. On the other hand, OGG1 can promote DNA demethylation at the sites of 8-oxoG by recruiting TET1/TET2 and reducing the DNMT1 occupancy [97,133]. After oxidative stress, TET1 is found in BER complexes together with APE1 and DNA polymerase β but not with OGG1 [129]; however, this co-occurrence may simply reflect TDG-dependent BER after the initiation of active demethylation by TET1.

Yet another option for 8-oxoG-triggered DNA demethylation could be a combination of long-patch BER and non-canonical mismatch repair, as observed for another oxidative lesion, 5-hydroxymethyluracil [63]. The repair of 8-oxoG in mCp(8-oxoG) dinucleotides in vitro is apparently normal and does not lead to the removal of mC immediately 5′ of 8-oxoG [168,169,170] but the possibility of mC erasure through extensive nicked strand degradation cannot be ruled out. Direct excision of mC by MUTYH in the *IL2* promoter was also reported [171] but has not been confirmed independently so far.

### 3.5. Free 8-oxoG and 8-oxoGTP Can Act as Secondary Messengers

Another scantly studied way of participation of 8-oxoG and OGG1 in the regulation of cellular processes concerns a possible role of 8-oxoG as a secondary messenger. After the excision of 8-oxoG, it remains bound in the enzyme’s active site and acts as a general base to promote the β-elimination step of the reaction [172]. The kinetics of OGG1/8-oxoG dissociation after the release of DNA has not been studied but there are reports in the literature that the OGG1/8-oxoG complex can act as a guanine nucleotide exchange factor and thus activate small GTPases Ras, Rac, and Rho [173,174,175,176,177]. Cell treatment with free 8-oxoG base upregulates over 2000 transcripts involved in a number of processes including inflammatory response and dendritic cell activation [178,179]. It is possible that activation of Ras in this case can indirectly affect transcription via the canonical MAP kinase pathway. The question of how the OGG1/8-oxoG complex formed in the nucleus reaches the cytoplasm where small GTPases are localized remains open.

Since many cellular processes are regulated by guanine nucleotides, oxidation of the nucleotide pool might also be a signal of oxidative stress. Of small regulatory GTPases, 8-oxoGTP hyperactivates the Ras-ERK pathway but inhibits Rac and Cdc42 [180,181]. 8-oxoGTP also inhibits two key enzymes of the NO-dependent cGMP signaling pathway: guanylate cyclase and GTP cyclohydrolase 1 [182,183].

### 3.6. Biological Function: 8-oxoG Is Involved in Inflammatory Response Regulation

Perhaps the hottest area of research into 8-oxoG-dependent regulation of gene activity in recent years has been the potential role of this DNA lesion in regulating the immune response. The advent of knockout mouse models has led to the discovery that OGG1 deficiency significantly reduces the intensity of the innate and adaptive immune response to a wide range of stimuli, including many known human allergens [95,106,179,184,185,186,187,188,189,190,191] (Table 2). Depletion or pharmacological inhibition of OGG1 in wild-type cells have the same effect [91,96,188,192,193,194]. Yet unchallenged, *Ogg1* knockout mice demonstrate many signs of metabolic syndrome, including adipose tissue inflammation, especially at advanced age or if fed high-fat diet [195,196,197,198].

Consistent with the above-described specificity of OGG1 interaction with transcription factors (see Section 3.1), the 8-oxoG/OGG1 mechanism seems to be most pronounced for the pro-inflammatory genes regulated through the TNFα/NF-κB pathway [84,91,92,93,206]. Moreover, OGG1 may be involved in other pathways dependent on NF-κB or its p65 subunit (RELA), such as metabolic reprograming in response to viral infection [207]. On the contrary, the pro-inflammatory pathways induced by O_2_ hyperoxia or acute exposure to UV light seems to be up-regulated in the absence of OGG1 despite ROS are believed to be the initiating factor in UV-induced inflammation [199,200,208,209,210,211]. In addition to the 8-oxoG/OGG1 signaling at the promoters of pro-inflammatory genes, induction of the inflammatory response enables a positive-feedback loop through STAT1 transcription factor, which induces *OGG1* gene expression, while OGG1 protein binds and co-activates STAT1 [189]. The pro-inflammatory action of 8-oxoG was also suggested to depend on Z-DNA formation. 8-oxoG is known to promote B-to-Z transition in GC-rich DNA duplexes [212,213], and potential Z-DNA-forming sequences are enriched in gene promoters with 8-oxoG and AP sites located within these sites modestly stimulating transcription from reporter plasmids [213]. Moreover, it has been shown that oxidized mitochondrial DNA released into cytoplasm easily adopts Z-form and is recognized by Z-DNA-binding protein (ZBP1), triggering innate immune response and neuroinflammation [214]. Notably, while the ability of OGG1 to bind and excise 8-oxoG from Z-DNA have not been studied, there is an abundant pool of OGG1 in the cytoplasm [215] so its participation in the extranuclear DNA-driven inflammation cannot be excluded.

Inflammatory responses not regulated by TNFα/NF-κB, such as the inflammasome cascade or interferon type I signaling, are either independent of or suppressed by OGG1 [216,217]. In particular, OGG1 interacting with sirtuin 3 dampens the response triggered by mitochondrial DNA oxidation [218,219,220,221]. However, in the case of the inflammasome, it was suggested that its key component, the NLRP3 protein, can specifically bind 8-oxoG in released damaged mitochondrial DNA for activation in an OGG1-independent manner [222,223]. Free 8-oxo-2′-deoxyguanosine also dampens the inflammatory response [224,225,226,227]. Extracellular mitochondrial DNA rich in unrepaired 8-oxoG also potently activates Toll-like receptor 9 (TLR9), eliciting dendritic cell response [228].

OGG1-initiated excision of 8-oxoG may also trigger inflammatory response through immunogenic cell death. Excessive removal of 8-oxoG under oxidative stress conditions leads to accumulation of single-strand DNA breaks, which activate poly(ADP-ribose)polymerase 1 (PARP1) and induce parthanatos, a special form of regulated cell death with elements of necrosis [229,230,231]. On the contrary, OGG1 protects cells from non-immunogenic apoptosis, mainly through its involvement in maintaining mitochondrial integrity [218,232,233].

**Figure 5:**
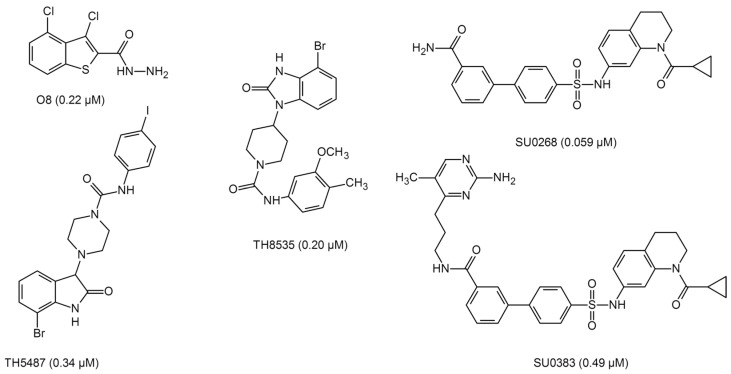
Structure of selected OGG1 inhibitors [192,234,235,236,238]. IC_50_ values are shown in parentheses.

Dependence of pro-inflammatory gene activation on OGG1 and 8-oxoG formation has led to the emergence of a model in which G oxidation and further reactions involving OGG1 are necessary to maintain a full-scale inflammatory response. In this regard, OGG1 is now viewed as a target for novel anti-inflammatory drugs, and several small molecules have been developed that are highly effective in both inhibiting OGG1 activity in vitro and reducing the production of pro-inflammatory cytokines in cell culture and in vivo [96,192,193,194,234,235,236,237,238,239] (Figure 5). However, since OGG1 is primarily required to protect the genome from damage and prevent mutations, the long-term safety of such agents is rather questionable, and the off-target effects are considerable [240].

## 4. Is 8-Oxoguanine Epigenetic?

As stated in Section 2, a bona fide epigenetic DNA label should be written and erased in a regulated manner, read by specific readers, and be non-mutagenic and non-genotoxic. Although there is plenty of mentions of 8-oxoG as an “epigenetic” label, this modified base does not meet at least two of these criteria.

### 4.1. 8-oxoG Is Pro-Mutagenic

The major problem with 8-oxoG being an epigenetic DNA label is of course its ambiguous coding properties, leading mainly to G→T transversions after the replication. Isolated mammalian replicative DNA polymerases α, δ and ε strongly prefer both to incorporate dAMP opposite 8-oxoG in the template and to extend dA(3′): 8-oxodG terminal primer pair [8,241,242,243,244]. However, the situation in more complete systems is not as simple. First, in the presence of other replication machinery components, such as replication protein A (RPA) and proliferating cell nuclear antigen (PCNA) this preference tips towards dCMP, although dAMP incorporation and extension is still considerable [8,241,243,244,245]. Second, and probably more important, 8-oxoG bypass is assisted by specialized translesion DNA polymerases. Of those, the best-studied with respect to 8-oxoG bypass are DNA polymerases η, ι and κ, as well as DNA polymerases β and λ, which are not specialized for translesion synthesis but still encounter 8-oxoG under some circumstances. Since translesion polymerases usually trade fidelity for the ability to bypass non-canonical bases, they may incorporate all four nucleotides opposite 8-oxoG with different efficiencies. Polκ prefers dAMP [246,247,248,249,250,251], Polι mostly incorporates dCMP but also a considerable amount of dGMP [243,248,251,252,253,254,255], whereas Polη efficiently bypasses 8-oxoG in an error-free manner [243,251,256,257,258,259]. Another translesion DNA polymerase, Polν, has not been characterized biochemically with respect to incorporation preference opposite 8-oxoG but its depletion in human cells alleviates G→T mutagenesis at a site-specific 8-oxoG adduct in a reporter plasmid, suggesting error-prone bypass [260]. Polλ has little dCMP/dAMP insertion bias but strongly prefers to extend the dC(3′):8-oxodG primer termini [243,244,261,262] and seems to be the first polymerase acting after MUTYH initiates the removal of A opposite 8-oxoG [263].

With this plethora of polymerases capable of error-prone and error-free 8-oxoG bypass, it is hardly surprising that this lesion is considerably mutagenic in mammalian cells. It should be kept in mind that most experiments in cellulo were carried out using reporter plasmids, and many of those used single-stranded or gapped plasmids to prevent repair, so the numbers may not reflect the situation of normal replication. In early studies with single-stranded phagemids carrying an SV40 origin, the mutagenicity level in COS7 cells (immortalized monkey kidney fibroblasts) was reported at 3–7% [264,265,266]. In contrast, a double-stranded plasmid with the same replication origin in the same cell line scored no mutations when carrying an 8-oxoG:C pair but yielded 36% mutant progeny from an 8-oxoG:A mispair [267] indicating that BER quickly removes 8-oxoG from the former context, but the full GO system works much more slowly. On the other hand, 8-oxoG in a single-stranded phagemid showed 10–15% mutagenicity in HEK293T human embryonic kidney cells [268] and nearly 100% mutagenicity in H1299 lung carcinoma cells [269,270]. A gapped plasmid with 8-oxoG in the 22-nt gap produced ~20% mutations in H1299 cells in a replication-independent manner [271].

With the advent of high-throughput sequencing it became possible to analyze spectra of mutations arising in their native conditions. In human cancers, single base substitution signatures SBS18 and SBS36 are attributed to 8-oxoG mutagenesis on the MUTYH-positive and -negative background, respectively [9,272,273,274,275,276]. Moreover, direct nucleotide-resolution mapping of the sites of 8-oxoG formation reveals high similarity with the SBS18/SBS36 sequence context, reinforcing the idea of 8-oxoG being the precursor of these mutations [277]. Interestingly, the SBS18 signature is correlated with regions of late replication, which are associated with heterochromatin and repressed genes, suggesting their less efficient repair [278]. In a rare example of 8-oxoG site-specifically introduced into the thymidine kinase gene in the BER-competent human lymphoblastoid TSCER122 cells through recombination with a synthetic donor, the mutagenicity was ~10% [279].

### 4.2. 8-oxoG Is Not Strictly Controllable

Epigenetic DNA labels are introduced by specific enzymes in a controllable manner, e.g., DNMT methyltransferases lay down mC labels, and TET dioxygenases convert them to hmC labels. So far, no such pathway has been discovered for 8-oxoG. Known DNA dioxygenases from the ten–eleven translocation (TET) and AlkB homolog (ALKBH) families do not process regular purine bases [280,281]. Examples of human enzymes oxidizing free purine bases or nucleotides at C8 do exist. The best-known one is xanthine dehydrogenase/oxidase, which converts free xanthine base to uric acid [282]. Aldehyde oxidase AOX1 participates in several purine drug metabolism pathways, for example, oxidizing free 6-thioguanine to 8-hydroxy-6-thioguanine or acyclovir to 8-hydroxyacyclovir [283,284]. Cytochromes P450, in particular CYP1A2 and CYP2A6, can oxidize some purine metabolites such as 1,7-dimethylxanthine, at C8 [285]. However, none of these enzymes or their homologs have been reported to process purines in DNA, and their cellular localization and/or multimeric structure make it unlikely that they can participate in targeted 8-oxoG generation.

In the absence of active G oxidation, the generation of 8-oxoG on DNA has to rely on spontaneous reactions. The key question here is whether the flux of ROS from any source will be sufficient to convert a considerable fraction of G in gene promoters to 8-oxoG to support robust gene activation. Take the case of LSD1, which catalyzes oxidative demethylation of H3K9 in two steps: first, *N*,*N*-dimethyllysine is oxidized to *N*-methyl-*N*-methylidenelysine, which is then hydrolyzed to *N*-methyllysine and formaldehyde [127,286]. FAD serves as an electron acceptor, and the resulting FADH^−^ is converted back to FAD with O_2_ as a possible oxidant in the step where H_2_O_2_ is generated. However, the reaction with O_2_ is rather slow (*k*_obs_ = 0.112 s^−1^ at atmospheric pressure and likely much less at the intracellular O_2_ concentrations in living tissues, which is about 1/10 of that [287]), raising questions about whether it is the physiologic terminal acceptor [127]. Given that H_2_O_2_ predominantly produces strand breaks rather than 8-oxoG [288,289], one may doubt that histone demethylation in situ is a reliable source of direct targeted oxidative DNA modification.

An appreciated but experimentally underexplored possibility to connect oxidative DNA damage with signaling is through charge migration in DNA. Hole conductivity is a salient feature of double-stranded DNA; once an electron is lost from a base or a deoxyribose, the hole can be delocalized through the base stack over a long distance (at least several hundred base pairs) until it reaches an easily oxidizable sink, which is usually the first or second G in a G-run [136,137,138,290]. Another type of sink is represented by DNA-binding proteins containing redox-sensitive cofactors, such as iron–sulfur (FeS) clusters [291,292]. In many cases, oxidation of the cluster greatly increases the protein’s affinity for DNA, so FeS-containing DNA repair proteins are suggested to use charge migration as means of remote sensing of oxidative DNA damage [293,294,295]. Hence, it is possible that 8-oxoG may be simply a marker of the final destination of electron vacancies, whereas the actual transcription activation may require oxidation of some regulatory proteins. While the human transcription factors responding to oxidative stress (Section 3.1) lack FeS clusters, these structural elements are found in the basal transcription machinery, e.g., in the XPD subunit of the TFIIH transcription factor and in RPC6, an RNA polymerase III regulatory subunit [296]. Intriguingly, the mitochondrial isoform of OGG1 acts as an oxidation protection factor for the FeS cluster in aconitase [232,233], suggesting a possibility that nuclear OGG1 might interact with FeS proteins, too. OGG1 itself possesses a redox-sensitive Cys253 residue in the active site [297] but it is not known whether it, or thiols in general, can trap holes migrating in DNA.

### 4.3. OGG1 Is a Dedicated Eraser, but Is It a Good Reader?

As mentioned in the Introduction, OGG1 is the main DNA glycosylase responsible for 8-oxoG removal in human cells. Direct measurements of 8-oxoG repair in the cell give the half-life values of several hours [298,299,300], providing an estimate of the time window for the regulatory action of this modified base. The half-life is much longer in OGG1-deficient cells [298,299,300], confirming the role of OGG1 as the 8-oxoG eraser. The current models of gene activation by 8-oxoG, however, imply that OGG1 is both the eraser and the reader. These two functions are obviously hard, albeit not impossible, to combine in a single protein, and the well-studied cytosine methylation system offers no example of such a hybrid effector.

The key point of discordance for OGG1 being a reader is the kinetics and thermodynamics of 8-oxoG recognition and excision. In a normal situation when OGG1 finds 8-oxoG in regular B-DNA, it quickly excises the lesion and is displaced by APE1 that nicks the AP site, so the whole process takes only minutes [23,25,26]. Slowly processed substrates, such as non-canonical DNA structures including G-quadruplexes, are either bound by OGG1 much less efficiently than B-DNA or show the same affinity for undamaged and damaged DNA [301,302]. So far the most reliable transcription activation scheme involves quick processing of 8-oxoG by OGG1 and APE1 and suppression of further repair, generating persistent single-strand breaks. Single-strand breaks are significantly enriched in open chromatin, active promoters and enhancers [303,304,305,306], although it is not known whether they contribute to these chromatin properties or are caused by them. Alternatively, base excision or DNA nicking may destabilize DNA and trigger conversion of PQSs to G-quadruplexes proper, which are further recognized by regulatory proteins [307]; in this case stable association of OGG1/APE1 with oxidized DNA is obviously not required (Figure 4c).

**Figure 6:**
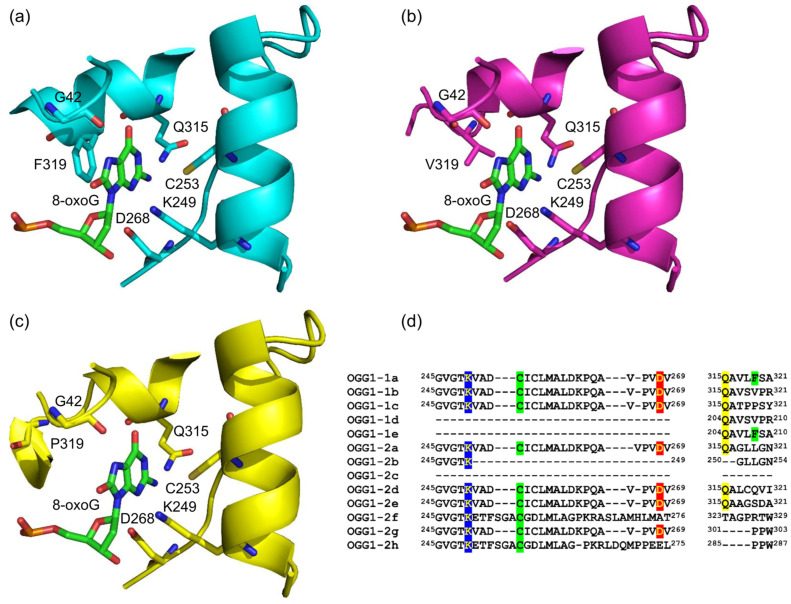
Active site pocket in various isoforms of OGG1. (**a**–**c**) View of the active sites in the models of OGG1-1a (**a**), OGG1-1b (**b**) and OGG1-1c (**c**). AlphaFold 3 [310] was used for structure prediction, and the top-ranking models were visualized with PyMol (Schrödinger, New York, NY, USA). (**d**) Alignment of the residues forming the active site in Group 1 (nuclear) and Group 2 (mitochondrial) OGG1 isoforms. The alignment was produced by Clustal Omega [311]. The highlighted residues form the catalytic dyad (Lys249 and Asp268; OGG1-1a numeration), recognize the Watson–Crick edge of 8-oxoG (Gln315) and stack with 8-oxoG in the active site (Cys253 and Phe319).

Another possible way to separate the reading and erasing functions of OGG1 is to engage an inactive enzyme as an 8-oxoG reader. As mentioned in Section 3.1, this might be achieved through oxidation of critical Cys residues in OGG1. Moreover, human cells express at least thirteen isoforms of OGG1, of which only one, OGG1-1a, is characterized biochemically [308]. Some of these isoforms possess a modified active site and could be involved in 8-oxoG reading without excision. In particular, there are five Group 1 isoforms, which are considered nuclear due to the presence of a nuclear localization signal. Of these, OGG1-1b and OGG1-1c have the active site conserved except for the C-terminal half of the αO helix, which is variable due to alternative splicing (Figure 6). In OGG1-1a, this part of the protein carries the Phe319 residue, which stacks against 8-oxoG in the active site [35]; the F319A mutant loses the ability to bind 8-oxoG-containing DNA [309] and to promote SMAD3 binding to the targeted promoters [97]. In OGG1-1b and OGG1-1c, this Phe is changed to Val and Pro, respectively (Figure 6), which might be better suited for 8-oxoG binding but prevent the enzymatic activity. This possibility still remains to be addressed experimentally.

Attempts to search for 8-oxoG binding proteins other than OGG1 in human cells so far produced rather controversial results. There were reports that 8-oxoG in dsDNA is bound with high affinity by the isolated ribosomal uS3 protein [312,313], while other studies found no difference in the uS3 binding to normal and damaged DNA [314]. In a mouse model of skin inflammation induced by 12-*O*-tetradecanoylphorbol-13-acetate, application of uS3 fused to a cell-penetrating peptide inhibited rather than promoted production of pro-inflammatory cytokines [315], suggesting that even if extra-ribosomal uS3 is an 8-oxoG reader, its effects are opposite to those of OGG1. Specific binding of 8-oxoG-containing DNA was reported for human single-strand binding protein 1 (SSB1) [316], AU-rich element RNA-binding protein 1 (AUF1) [317] and Y-box binding protein 1 (YBX1) [318]; however, the possible role of this binding has not been elucidated.

## 5. Conclusions

**Figure 7:**
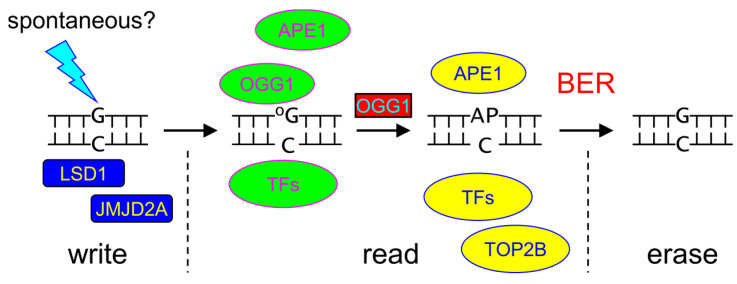
Current view of the write–read–erase cycle for gene activity regulation by 8-oxoG. TFs, transcription factors.

There is currently ample evidence that the role of 8-oxoG extends far beyond that of a mere pro-mutagenic DNA damage product to be swiftly eliminated; it is a key node in the cellular response to oxidative stress, particularly in orchestrating gene expression programs related to inflammation and adaptation. However, since the specific means of its generation are unknown, the mechanisms of its reading are not entirely clear, and 8-oxoG itself, unlike mC, has a high mutagenic potential, it cannot be considered an epigenetic base in the classical sense (Figure 7). Rather, 8-oxoG may be viewed as a peculiar signal of the cell’s oxidative status, unevenly distributed across DNA and triggering specific reactions when localized to certain genomic regions.

The case for 8-oxoG’s regulatory function is compelling (summarized in Table 3). Its accumulation in the promoters of specific genes, coupled with the demonstrated role of OGG1 in recruiting transcription factors such as NF-κB and HIF1α, provides a robust mechanism for targeted gene activation. This is further supported by pathways linking its formation to chromatin remodeling, where ROS generated by histone demethylases like LSD1 can locally produce 8-oxoG, creating a feed-forward loop for transcription. The enrichment of 8-oxoG in potential G-quadruplex-forming sequences in promoters adds another layer of topological control, where base excision and repair intermediates can facilitate structural transitions that influence transcription factor access. The profound impact of OGG1 inhibition on inflammatory responses underscores the physiological relevance of this 8-oxoG/OGG1 signaling axis.

Despite this, 8-oxoG fails to meet the core criteria for an epigenetic mark. First and foremost, it is unequivocally pro-mutagenic, posing a constant threat to genomic integrity. A true epigenetic mark must be faithfully inherited, a standard met by mC but definitively not by 8-oxoG. Second, its formation is not regulated in the manner of epigenetic writers. While 8-oxoG generation can be a consequence of regulated processes (e.g., histone demethylation), it is mostly produced stochastically by ROS or through charge migration in DNA, lacking specific ways to place it at definite genomic positions. This contrasts sharply with the targeted action of DNA methyltransferases. Finally, the system lacks dedicated reader proteins that interpret the mark without altering it. OGG1 itself is primarily an eraser. Its function in transcription often relies on its catalytic activity to create a repair intermediate (an AP site or a single-strand break) or on its transient, pre-excision binding. The models requiring a persistently bound OGG1 are kinetically challenging due to the rapid displacement by APE1. While enzymatically inactive OGG1 isoforms or oxidatively inactivated OGG1 could in principle serve as pure readers, this mechanism remains to be proven.

In conclusion, 8-oxoG is best understood as a redox-sensitive regulatory lesion. It acts as a powerful, dynamic signal for acute transcriptional responses to oxidative stress, functioning through sophisticated mechanisms involving OGG1-mediated recruitment of transcription machinery and alterations to DNA topology. 8-oxoG represents a fascinating interface between DNA repair and gene regulation but is not a stable carrier of heritable epigenetic information. Future research should focus on elucidating the precise structural complexes at oxidized promoters and exploring the therapeutic implications of modulating the 8-oxoG/OGG1 axis in inflammatory diseases, while acknowledging the inherent genomic risks of manipulating a fundamental DNA repair pathway.

## Figures and Tables

**Table 1:** Known and proposed epigenetic and regulatory DNA modifications in mammalian cells *.

Modification	Writers	Readers	Erasers
5-methylcytosine	DNMT1, DNMT3A, DNMT3B	MeCP2, MBD1, MBD2, MBD3, SETDB1, SETDB2, BAZ2A	TET1, TET2, TET3, then TDG
5-hydroxymethylcytosine	TET1, TET2, TET3	UHRF2, CHTOP	TET1, TET2, TET3, then TDG
5-hydroxymethyluracil	AID, APOBEC1, APOBEC2, APOBEC3A, APOBEC3C, APOBEC3E, TET1, TET2, TET3	MeCP2, non-canonical MMR	TDG, MBD4
6-methyladenine	N6AMT1, METTL3-METTL14, METTL4	ASXL1, MPND, YTHDC1	ALKBH1, ALKBH4
8-oxoguanine	Indirect: LSD1, JMJD2A; spontaneous?	OGG1, APE1, TOP2B	OGG1, APE1

* See main text for references.

**Table 2:** Inflammation-related phenotypes of *Ogg1* knockout mice and pharmacologic mouse models.

Phenotype	References
*Knockouts*	
Resistance to LPS- and oxazolone-induced inflammation; resistance to streptozotocin-induced type I diabetes	[184]
Resistance to Helicobacter pylori-induced gastric inflammation	[185]
Aggravated Pseudomonas aeruginosa-induced lung injury	[186]
Reduced allergic airway inflammation after sensitization and challenge by ovalbumin	[187]
Reduced allergic airway inflammation after sensitization and challenge by Ambrosia pollen grain extract	[188,190]
Aggravated lung inflammation after pulmonary hyperoxia	[199]
Aggravated UV-induced skin inflammation	[200]
Metabolic syndrome phenotype	[195,196,197,198]
Aggravated pulmonary fibrosis upon asbestos exposure	[201]
Reduced pulmonary fibrosis upon bleomycin treatment	[202]
Reduced pulmonary fibrosis upon TNFα treatment	[91]
Dextran sulfate-induced intestinal inflammation; increased population of pro-inflammatory bacteria in the gut microbiome	[203]
Enhanced microglia activation in Alzheimer’s disease model	[204,205]
Aggravated diabetic cardiomyopathy	[99]
***Pharmacological OGG1 inhibition***	
Reduced lung inflammation upon TNFα treatment	[192]
Reduced lung inflammation in respiratory syncytial virus infection	[106]
Reduced pulmonary fibrosis upon bleomycin treatment	[96]
Reduced pulmonary fibrosis upon TGFβ treatment	[95]
Reduced allergic airway inflammation after sensitization and challenge by ovalbumin	[194]
Reduced pancreatic injury upon cerulein treatment	[193]

**Table 3:** Summary of the effects of 8-oxoG on gene transcription.

Gene	TF *	Proposed Mechanism	References
*Activation*			
*B2M*	NF-κB	OGG1→TF recruitment	[84]
*BACE1*	unknown	OGG1→TET1 recruitment→DNA demethylation	[133]
*BCL2*	ERα	LSD1→OGG1→APE1→TOP2B→chromatin remodeling	[81]
*CAD*	Myc	LSD1→OGG1→APE1→TF recruitment	[130]
*CCL2*	NF-κB	OGG1→TF recruitment	[93,107]
*CCL3*	NF-κB	OGG1→Ras activation	[176]
*CCL5*	NF-κB	OGG1→TF recruitment	[93,106]
*CCL20*	NF-κB	OGG1→TF recruitment	[84,91,93,106,107]
	NF-κB	OGG1→Ras activation	[176]
*CDH2*	SMAD2/3	LSD1/JMJD2A→OGG1→TF recruitment	[131]
*COL1A1*	SMAD2/3/4	OGG1→TF recruitment	[95,96]
*CXCL1*	NF-κB	OGG1→TF recruitment	[84,91,93,107]
	NF-κB	OGG1→Ras activation	[176]
*CXCL2*	NF-κB	OGG1→TF recruitment	[91,92,93,102,107]
*CXCL8*	NF-κB	OGG1→TF recruitment	[93]
*CXCL10*	NF-κB	OGG1→TF recruitment	[106]
*FN1*	SMAD2/3/4	OGG1→TF recruitment	[95,96]
*FOXP3*	SMAD2/3/4	OGG1→TF and TET1/2 recruitment, DNA demethylation, decreasing DNMT1 occupancy	[97]
*IL1A*	NF-κB	OGG1→TF recruitment	[91]
	NF-κB	OGG1→Ras activation	[176]
*IL1B*	NF-κB	OGG1→TF recruitment	[91,102]
	NF-κB	OGG1→Ras activation	[176]
*IL6*	NF-κB, NRF1	OGG1→APE1→TF recruitment	[106,107,118]
*IL8*	NF-κB, NRF1	OGG1→APE1→TF recruitment	[90,118]
*IL10*	NF-κB, NRF1	OGG1→APE1→TF recruitment	[118]
*KLK3*	AR	LSD1→OGG1→APE1→TOP2B→chromatin remodeling	[128]
*KRAS*	MAZ	OGG1→APE1→quadruplex refolding	[98,148]
*miR-125b2*	AR	LSD1→OGG1→APE1→TOP2B→chromatin remodeling	[128]
*miR-133b*	AR	LSD1→OGG1→APE1→TOP2B→chromatin remodeling	[128]
*MYC*	YY1	OGG1→PRMT1→chromatin remodeling	[100]
	unknown	OGG1→APE1→quadruplex refolding	[148]
*NCL*	Myc	LSD1→OGG1→APE1→TF recruitment	[130]
*NTHL1*	APE1(?)	OGG1→APE1→quadruplex refolding	[141]
*PAI*	SMAD2/3	LSD1/JMJD2A→OGG1→TF recruitment	[131]
*PCNA*	APE1(?)	OGG1→APE1→quadruplex refolding	[142]
*PUMA*	Sp1	TF enhanced binding	[86]
*RARB*	RAR	LSD1→OGG1(?)→APE1→chromatin remodeling	[129]
*SIRT1*	APE1(?)	OGG1→APE1	[82]
*SNAI1*	SMAD2/3	LSD1/JMJD2A→OGG1→TF recruitment	[131]
*TMPRSS2*	AR	LSD1→OGG1→APE1→TOP2B→chromatin remodeling	[128]
*TNF*	NF-κB, NRF1	OGG1→APE1→TF recruitment	[84,91,93,102,106,107,118]
	NF-κB	OGG1→Ras activation	[176]
*TNIP1*	NF-κB	TF enhanced binding	[90]
*VEGF*	Hif-1α	OGG1→APE1→TF recruitment	[83]
	Hif-1α, APE1(?)	OGG1→APE1→quadruplex refolding	[140,141,148]
	Sp1	TF enhanced binding	[80]
*VIM*	SMAD2/3/4	LSD1/JMJD2A→OGG1→TF recruitment	[95,96,131]
***Repression***			
*ACTB*	unknown	OGG1→SIRT1, EZH2, DNMT1/3B recruitment, chromatin remodeling	[167]
*CDH1*	SNAI1	OGG1→CHD4, EZH2, DNMT1/3A/3B recruitment, chromatin remodeling	[34,166]
*CDKN2A*	unknown	OGG1→CHD4, EZH2, DNMT1/3A/3B recruitment, chromatin remodeling	[34]
*IFNL2*	NF-κB1	OGG1→transcription repressor recruitment	[119]
*IFNL3*	NF-κB1	OGG1→transcription repressor recruitment	[119]
*MLH1*	unknown	OGG1→CHD4, EZH2, DNMT1/3A/3B recruitment, chromatin remodeling	[34,167]
*MYC*	unknown	OGG1→SIRT1, EZH2, DNMT1/3B recruitment, chromatin remodeling	[167]
*RAD17*	unknown	quadruplex refolding	[152]
*SFRP4*	unknown	OGG1→CHD4, EZH2, DNMT1/3A/3B recruitment, chromatin remodeling	[34]
*SFRP5*	unknown	OGG1→CHD4, EZH2, DNMT1/3A/3B recruitment, chromatin remodeling	[34]
*TIMP2*	unknown	OGG1→CHD4, EZH2, DNMT1/3A/3B recruitment, chromatin remodeling	[34]
*TIMP3*	unknown	OGG1→CHD4, EZH2, DNMT1/3A/3B recruitment, chromatin remodeling	[34,167]
*VEGF*	Sp1	quadruplex refolding, TF eviction	[150]
*WIF1*	unknown	OGG1→CHD4, EZH2, DNMT1/3A/3B recruitment, chromatin remodeling	[34]
TF binding elements only	CREB	OGG1 binding interferes with TF binding	[120]
	Sp1	OGG1→APE1 interferes with TF binding	[123]
GFP reporter	gene body	unprocessed 8-oxoG or OGG1→APE1 interferes with transcription elongation complex	[121,122,124]
		histone deacetylation, chromatin remodeling	[126]

* TF, transcription factor(s).

## Data Availability

No new data were created or analyzed in this study. Data sharing is not applicable to this article.
